# Multi-site study of HPV type-specific prevalence in women with cervical cancer, intraepithelial neoplasia and normal cytology, in England

**DOI:** 10.1038/sj.bjc.6605747

**Published:** 2010-07-13

**Authors:** R Howell-Jones, A Bailey, S Beddows, A Sargent, N de Silva, G Wilson, J Anton, T Nichols, K Soldan, H Kitchener

**Affiliations:** 1 HIV & STI Department, Health Protection Agency Centre for Infections, 61 Colindale Avenue, London NW9 5EQ, UK; 2 Department of Clinical Virology, Central Manchester NHS Foundation Trust, Second Floor Clinical Sciences Building 2, Manchester Royal Infirmary, Oxford Road, Manchester M13 9WL, UK; 3 Virus Reference Department, Health Protection Agency Centre for Infections, 61 Colindale Avenue, London NW9 5EQ, UK; 4 Department of Histopathology, Central Manchester NHS Foundation Trust, Second Floor Clinical Sciences Building 2, Manchester Royal Infirmary, Oxford Road, Manchester M13 9WL, UK; 5 Statistics, Modelling and Bioinformatics Department, Health Protection Agency Centre for Infections, 61 Colindale Avenue, London NW9 5EQ, UK; 6 School of Cancer and Enabling Sciences, University of Manchester, Manchester M13 9WL, UK

**Keywords:** human papillomavirus (HPV), cervical cancer, cervical screening, immunisation

## Abstract

**Background:**

Knowledge of the prevalence of type-specific human papillomavirus (HPV) infections is necessary to predict the expected, and to monitor the actual, impact of HPV immunisation and to design effective screening strategies for vaccinated populations.

**Methods:**

Residual specimens of cervical cytology (*N*=4719), CIN3/CGIN and cervical cancer biopsies (*N*=1515) were obtained from sites throughout England, anonymised and tested for HPV DNA using the Linear Array typing system (Roche).

**Results:**

The prevalence of HPV 16 and/or 18 (with or without another high-risk (HR) type) was 76% in squamous cell carcinomas, 82% in adeno/adenosquamous carcinomas and 63% and 91% in CIN3 and CGIN, respectively. Of all HR HPV-infected women undergoing cytology, non-vaccine HPV types only were found in over 60% of those with mild dyskaryosis or below, and in <20% of those with cancer. In women of all ages undergoing screening, HR HPV prevalence was 16% and HPV 16 and/or 18 prevalence was 5%.

**Conclusion:**

Pre-immunisation, high-grade cervical disease in England was predominantly associated with HPV 16 and/or 18, which promises a high impact from HPV immunisation in due course. Second-generation vaccines and screening strategies need to consider the best ways to detect and prevent disease due to the remaining HR HPV types.

A wide range of international studies have found approximately 70% of cervical cancers to be associated with human papillomavirus (HPV) types 16 or 18, and therefore preventable by currently available HPV vaccines (Clifford *et al*, 2003; Muñoz *et al*, 2004). The United Kingdom introduced an HPV immunisation programme in September 2008 and expects to see a consequent decline in cervical cancers and cervical intraepithelial neoplasia in the coming decades (Choi *et al*, 2009).

In 2006, almost 3000 women were diagnosed with cervical cancer in the United Kingdom, and there were around 1000 deaths (Cancer Research UK, 2009). It has been estimated that cervical screening prevents at least 70% of cases that would occur without screening (Sasieni *et al*, 2003). To achieve this, the NHS Cervical Screening Programme (NHS CSP) in England screens more than 3 million women aged 25–64 years annually and refers more than 100 000 to colposcopy examination (The NHS Information Centre, 2009).

Several studies have investigated HPV prevalence in women attending for cervical screening in the United Kingdom (Cuschieri *et al*, 2004; Kitchener *et al*, 2006; Hibbitts *et al*, 2008) and have provided valuable data regarding the frequency of HPV infection. These studies have been conducted in specific areas of the country, at different times, using different testing methods, and as they have sampled all women attending for screening they have inevitably provided fewer data regarding the rarer disease-grades, and some age-groups.

Better knowledge of HPV type-specific prevalence and distribution at each stage of cervical disease and among women of different ages would both improve estimates of the expected impact from HPV 16/18 immunisation and inform planning of possible future screening services, particularly those using HPV testing.

The aim of this study was to provide robust baseline estimates of the prevalence and distribution of HPV types among a nationally representative sample of women, for the full age range undergoing screening (25–64 years) and for each disease phase (from normal cytology to cervical cancer), prior to any impact of the National HPV Immunisation Programme.

## Materials and methods

### Sample collection and processing

Two sample types were included in the study: (i) tissue sections from routinely obtained diagnostic biopsies of cervical cancers and high-grade precancerous lesions archived in NHS pathology laboratories, and (ii) residual liquid-based cytology (LBC) samples from women attending for cervical screening. Samples were obtained from eight participating NHS pathology laboratories in England. Three laboratories (Gateshead Health NHS Foundation Trust, Birmingham Women’s NHS Foundation Trust and Royal Free Hampstead NHS Trust (London)) provided both tissue sections and LBC samples, three laboratories (Central Manchester University Hospitals NHS Foundation Trust, Sheffield Teaching Hospitals NHS Foundation Trust and Barts and The London NHS Trust) provided tissue sections only and two laboratories (Gloucestershire Hospitals NHS Foundation Trust and Norfolk and Norwich University Hospitals NHS Foundation Trust) provided LBC samples only.

*Cancer and CIN3/CGIN biopsies* Sections from archived blocks of cervical intraepithelial neoplasia 3 (CIN3), cervical glandular intraepithelial neoplasia (CGIN), squamous cell carcinoma (SCC) and adenocarcinoma (including adeno-squamous carcinoma) (ADC) tissue were collected from the six participating sites between June 2006 and July 2008. Cases were deemed suitable for inclusion in the study after confirmation of the pathological diagnosis by an experienced subspecialist gynaecological histopathologist at each of the six participating centres. The biopsies of cancer had been originally embedded between 1986 and 2008; 96% had been embedded since 2000. The CIN3 and CGIN biopsies had been collected between 2000 and 2008. Approximately six 10 μ thick sections from each tissue block were placed in a 1.6 ml Eppendorf tube. A further section was stained and re-examined by the pathologist to confirm the presence of diseased tissue. To prevent cross-contamination from residual wax from the previous block, the microtome blade was cleaned with ethanol between blocks. Tissue sections were sent to the Department of Clinical Virology, Central Manchester University Hospitals NHS Foundation Trust for HPV genotyping. The following data were collected on a study record for each sample and sent to Manchester: month and year of birth, date of sample collection, type of lesion (CIN3, CGIN or cancer) and histology result.

Tissue sections were de-waxed by adding a 1 ml of octane to each Eppendorf tube followed by 75 *μ*l of methanol. Tubes were vortexed and incubated at 56 °C for 30 min followed by centrifugation at 13 000 r.p.m. for 1 min after which the octane layer was removed using a fine-tipped Pasteur pipette. The tissue pellet was then washed with 1.0 ml of ethanol, centrifuged as before and the ethanol removed. The tubes were then left at 56 °C for 30–45 min to evaporate off residual ethanol. The tissue was digested in 600–1000 *μ*l (depending on size of pellet) of proteinase K lysis buffer containing 10 mM Tris/HCl buffer pH 8.3; 1 mM EDTA, 0.5% Triton X-100, 0.002% SDS and 250 *μ*g ml^–1^ of proteinase K. Tissue digestion was carried out at 56 °C for 72 h with constant agitation. DNA was then extracted from a 200 μl aliquot of digested tissue using the Roche MagNA Pure (Roche Applied Science, Penzberg, Germany) automated extraction system with an elution volume of 100 *μ*l.

*LBC samples* Residual LBC samples were collected prospectively from women undergoing cervical screening between October 2007 and January 2009 at five participating sites. Stratified sampling was used to obtain sufficient high-risk (HR) HPV-positive samples to analyse type distribution within each combination of age-band and cytology grade, thus a disproportionately higher number of older women and those with more severe cytology outcomes were sampled. Before inclusion in the study, samples were collected and handled according to local protocols: Thinprep and Surepath LBC systems were both in use, with samples stored at ambient temperatures while awaiting cytological examination. After completion of cytology, residual LBC samples that matched an unmet age-group and grade (according to the site’s stratified sampling frame) were anonymised and sent to the Health Protection Agency (HPA) Virus Reference Department for HPV genotyping. For Surepath samples, the tubes (enriched sample), not the vials, were used as the vials were found to have an inadequate amount of cellular material. The tube approximated to levels found using Thinprep, although with more variability. The following data were collected on a study record for each sample and sent to the HPA: month and year of birth, cytology result, date of sample, outward postcode (i.e., up to the first four characters), biopsy taken (and histology result if applicable).

1 ml of each LBC sample was centrifuged for 5 min at 13 000 r.p.m. and the cellular pellet suspended in 300 *μ*l of cold sterile phosphate-buffered saline. After a lysis step (addition of 40 *μ*l Qiagen Protease and 265 *μ*l Qiagen Buffer AL containing guanidine hydrochloride) samples were stored at −25 °C before nucleic acid extraction. Nucleic acid extraction was conducted on the BioRobot Universal platform (Qiagen, Crawley, West Sussex, UK) using the QIAamp DNA Blood BioRobot MDx kit and the extraction protocol QIAamp ‘One for All UNIV rcV23’.

### HPV Genotyping

A 50 *μ*l aliquot of the extracted DNA from the biopsy samples and a 10 *μ*l aliquot of the extracted DNA from the LBC samples (i.e., approximately 10% of the original 1 ml material, equal to the suggested input for the Roche Linear Array (LA) test (Roche Molecular Systems, Inc., Branchburg, NJ, USA)) was used for the PCR reaction stage of the Roche LA test. The Roche LA test was used according to manufacturer’s instructions, with the exception of the sample extraction methods described above. Individual oligonucleotide capture probes enabled identification of 37 HR and low-risk HPV genotypes and the *β*-globin amplicon acted as a control for cell adequacy, extraction and amplification. Identification of HPV 52 in this system was possible by the use of a cross-reactive oligonucleotide probe (52 M) that hybridises with HPV genotypes 33, 35, 52 and 58. The presence of HPV 52 was therefore inferred by reactivity to the 52 M probe with no reactivity to the individual oligonucleotide probes representing HPV 33, 35 and 58. The presence of a co-infection of 52 with 33, 35 or 58 would not have identified HPV 52. The HPV-positive and -negative controls provided within the Roche LA test kit were included for every 46 tissue section samples and every 22 LBC samples tested. The time from LBC sample collection to receipt at HPA (median 27 days, inter-quartile range 21–35) was not negatively associated with detection of *β*-globin or HPV DNA: this variable was not considered further.

Following an assessment of type-specific assay reproducibility (details in Supplementary online material) only those bands that gave a signal equal to or greater than the *β*-globin low control band were recorded.

**Figure 1:**
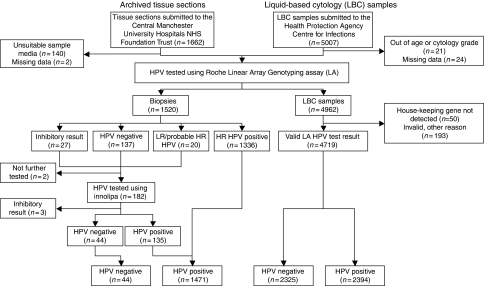
Sample submission and human papillomavirus (HPV) testing algorithm for archived tissue sections and residual liquid based cytology (LBC) samples. Abbreviations: HR=high-risk; LR=low-risk.

For tissue section samples only, additional analysis using the Inno-Lipa Extra (Microgen Bioproducts Ltd, Camberley, Surrey, UK) genotyping assay was performed on those samples that were *β*-globin negative or in which no HR HPV DNA was detected using Roche LA test (Figure 1). The Inno-Lipa assay is a similar format line blot assay to Roche LA (and also includes a *β*-globin control), however, it amplifies a much smaller fragment of the DNA than the Roche LA assay making it less likely to be affected by DNA fragmentation, which may occur during tissue fixation. A 5 *μ*l aliquot of extracted DNA was amplified and detected according to manufacturer’s instructions.

Figure 1 shows the flow of tissue sections and LBC samples included in the study.

### Data analysis

A valid result was defined as any sample with a positive *β*-globin result by either Roche LA or Inno-Lipa Extra. Only samples with a valid HPV typing result (*N*=6234, Figure 1) were included in the analysis. HR HPV types were defined according to the 2009 International Agency Research on Cancer classification of types, which were at least ‘probably carcinogenic to humans’, that is, HPV 16, 18, 31, 33, 35, 39, 45, 51, 52, 56, 58, 59 and 68 (Bouvard *et al*, 2009). Five groups of HPV types were defined for the purpose of the analysis: (i) HPV 16 and/or HPV 18 irrespective of other types, (ii) HPV 16 and/or 18 without any other HR types, (iii) HPV 16 and/or 18 with other HR type(s) (iv) HR HPV (any one or more type(s)) but not HPV 16 or HPV 18 and (v) any HR HPV infection. The prevalence of HPV infection, for each group above, for each age-group and disease-grade, was calculated with 95% confidence intervals (CIs) that allowed for the samples being effectively clustered through the collection from a sample of laboratories. For the LBC samples, prevalence estimates, other than those for a particular age-band and cytology grade, were calculated using sampling weights based on the number of women attending for cervical screening as part of the NHS CSP in 2007–2008. This was necessary to estimate prevalence for the screened population as our stratified sampling purposely selected disproportionate numbers from each age-band and cytology-grade. To analyse prevalence in each region, sampling weights (based on national cervical screening data as described above) were used and applied to samples from each laboratory separately. Differences in prevalence by region were assessed using Pearson’s *χ*^2^ statistic.

## Results

Valid data and HPV results were obtained for 1515 biopsy sections and 4719 LBC samples submitted to the study. The biopsy sections consisted of 906 CIN3, 450 SCC, 54 CGIN and 105 ADC samples (including 89 adeno- and 16 adenosquamous-carcinomas), while the LBC samples were from 2452 women without cervical disease, 1051 with borderline, 697 with mild, 276 with moderate and 243 with severe dyskaryosis. The mean (s.d.) age of the women at biopsy was 31.9 (8.1), 49.7 (16.1), 35.4 (7.8) and 45.5 (14.1) years for CIN3, SCC, CGIN and ADC, respectively. The numbers of samples included in the study, by participating site, are available in Supplementary Table S1.

### HR and HPV 16 and/or 18 prevalence

**Figure 2:**
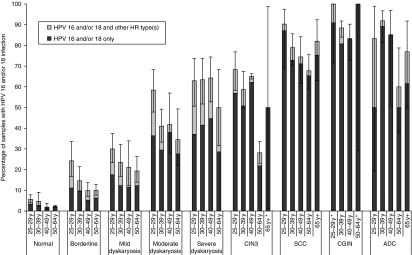
Prevalence of human papillomavirus (HPV) 16 and/or 18 (alone and in mixed infections with other high-risk (HR) HPV types) by cervical grade and age band. 95% confidence intervals were calculated allowing for samples being clustered within laboratories, except where these could not be determined because of there being no variation between laboratories (i.e. all 100% prevalence) or when all samples were from one laboratory (i.e. CIN3 samples from women aged 65+years), when confidence intervals (one-sided 97.5% where appropriate) were calculated without allowing for clustering (^*^). Abbreviations: ADC=adeno- and adeno-squamous carcinoma; CIN3=cervical intraepithelial neoplasia 3; CGIN=cervical glandular intraepithelial neoplasia; SCC=squamous cell carcinoma.

The prevalence of each of our defined categories of HR HPV, by age-group and disease-grade, from normal to cancer, are shown in Table 1. Figure 2 shows HPV 16 and/or 18 infections by disease-grade in each age-band, while additional data by disease-grade in each age-band are available in Supplementary Table S2. HR HPV types were detected in 95.8% (95% CI 94.4–96.8%) and 95.2% (95% CI 84.8–98.6%) of SCC and ADC, respectively. HPV 16 and/or 18 (alone or with other HR types) were detected in 76.4% (95% CI 71.0–81.1%) and 81.9% (95% CI 73.2–88.2%) of SCC and ADC, respectively, while HPV 16 and/or 18 were the only HR types detected in 72.0% (95% CI 67.3–76.3%) and 75.2% (95% CI 64.3–83.7%) of SCC and ADC, respectively (Table 1).

In biopsies from high-grade cervical abnormalities, HR HPV were detected in 94.2% (95% CI 87.7–97.3%) and 98.1% (95% CI 86.4–99.8%) of CIN3 and CGIN, respectively. HPV 16 and/or 18 were detected in 63.2% (95% CI 57.0–69.1%) and 90.7% (95% CI 85.0–94.4%) of CIN3 and CGIN, respectively, and were the only HR types detected in 53.9% (95% CI 48.7–59.0%) and 83.3% (95% CI 61.1–94.1%), of CIN3 and CGIN, respectively (Table 1).

**Figure 3:**
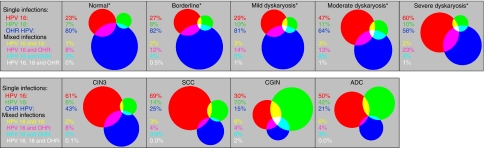
Proportional Venn diagrams showing human papillomavirus (HPV) 16, HPV 18 and high-risk (HR) types other than HPV 16 or HPV 18 (OHR) in HR HPV-positive samples, by cervical grade (Chow and Rodgers, 2005). Red: HPV 16; green: HPV 18; blue: OHR; yellow: HPV 16 and HPV 18; pink: HPV 16 and OHR; turquoise: HPV 18 and OHR; white: HPV 16, 18 and OHR. ^*^Age-weighted percentages (to allow for disproportionate liquid-based cytology sample collection by age). Abbreviations: ADC=adeno- and adeno-squamous carcinoma; CIN3=cervical intraepithelial neoplasia 3; CGIN=cervical glandular intraepithelial neoplasia; SCC=squamous cell carcinoma.

In women of all ages undergoing screening, the prevalence of HR HPV and of HPV 16 and/or 18 was 15.7% (95% CI 11.2–21.6%) and 5.1% (95% CI 3.3–8.0%), respectively (Table 1). The prevalence of HR HPV and HPV 16 and/or 18 increased with increasing severity of cytology grade, and was higher among younger women, within all cytology grades (more markedly in the lower grades) (Figure 2). Among residual LBC samples from women with severe dyskaryosis the prevalence of HR HPV ranged from 86% in 50- to 64-year olds to 97% in 25- to 29-year olds. The prevalence of HPV 16 and/or 18 in severe dyskaryosis alone or with other HR types showed no strong trend with age, at between 50 and 64%, with HPV 16 and/or 18 alone (without other HR types) detected in between 29 and 45% (Figure 2). The crude prevalence of HR HPV and of HPV 16 and/or 18 infection in the LBC samples from women confirmed on histology examination to have CIN3 disease was 95.0% (*n*=226) and 62.6% (*n*=149), respectively. Women of all ages with moderate dyskaryosis also had high prevalence of HR HPV and of HPV 16 and/or 18 infection (69–94% and 35–58%, respectively) (Figure 2 and Supplementary Table S2). With increasing disease-grade, there was an increasing proportion of HPV 16 and HPV 18 in HPV-positive samples, and a concomitant decrease in other HR HPV types (Figure 3).

Prevalence of HR HPV by region ranged from 13.3% in Gloucestershire to 20.1% in London, while prevalence of HPV 16 and/or 18 ranged from 4.2% in Gateshead to 6.5% in London (Table 2). Differences in prevalence by region were not statistically significant (Pearson's *χ*^2^ *P*>0.05). Further exploration of HR HPV prevalence in young women (25–29 years) with normal cytology results also found no significant differences between regions (data not shown).

### Type-specific prevalence

**Figure 4:**
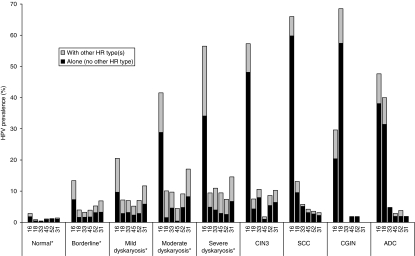
Human papillomavirus (HPV) prevalence by cervical grade of the six most common types found in squamous cell carcinoma (SCC) biopsies. ^*^Age-weighted prevalence (to allow for disproportionate liquid-based cytology sample collection by age). Abbreviations: ADC=adeno- and adeno-squamous carcinoma; CIN3=cervical intraepithelial neoplasia 3; CGIN=cervical glandular intraepithelial neoplasia.

HPV 16 was the most commonly detected type in all cervical grades apart from CGIN wherein HPV 18 was the most commonly detected type. The four next most frequently identified HPV types in the SCC biopsy samples (in single or multiple infections) were, in descending order, HPV 33, 45, 52 and 31. The prevalence of these six types in cancer, precancerous lesions and normal cytology samples is shown in Figure 4. The six most common HPV infections in women with normal cytology were, in descending order, HPV 16, 61, 62, 53, CP6108 and 54; HPV 18 was the nineteenth most common type (HPV type-specific prevalence by grade of disease is available in Supplementary Table S3).

The four HR types found most commonly in SCC biopsy samples, after HPV 16 and 18, showed a general pattern of increasing prevalence with increasing grade of disease although all, except HPV 45, had higher prevalence in severe grade lesions and CIN3 than in cervical cancers (Figure 4). The age-weighted prevalence of HPV 31 and 52 peaked in samples with moderate dyskaryosis (Figure 4).

The prevalence, in the absence of HPV 16 or 18, in SCC and ADC of selected HPV types from the A9 and A7 species, closely related to HPV 16 and 18 respectively, is shown in Table 3. The five types with emerging evidence of cross-protection from HPV 16/18 vaccines (HPV 31, 33, 45, 52 or 58) were found in 14.7% of SCC and 10.5% of ADC in the absence of HPV 16 and 18.

HPV 6 and/or 11 were detected in very few LBC samples without HR types (*n*=46, 0.8% weighted prevalence). Of these samples, 34.8% (*n*=16) had normal cytology, 30.4% (*n*=14) had borderline and 34.8% (*n*=16) had mild dyskaryosis. No women with moderate or severe dyskaryosis were found to have HPV 6 and/or 11 without a HR HPV type. Overall (irrespective of other types), the age-weighted (to NHS CSP population) prevalence of HPV 6 and/or 11 was 0.9% (95% CI 0.6–1.5%) in normal samples, 0.1% (95% CI 0.08–0.14%) in borderline samples, 0.1% (95% CI 0.05–0.3%) in mild dyskaryotic samples, 0.02% (95% CI 0.01, 0.1%) in moderate dyskaryotic and 0.01% (95% CI 0.0, 0.04%) in severe dyskaryotic samples. In women with normal cytology, the weighted prevalence of HPV 6 was 0.8% and HPV 11 was 0.2% (Supplementary Table S3).

## Discussion

We have used anonymous, residual, cervical cytology (LBC) samples and biopsies of cervical cancers and high-grade lesions, collected from eight sites, to describe type-specific HPV prevalence in women in England, across the full spectrum of cervical pathology, from normal to cancer.

Assuming the relative frequency of SCC and ADC in our study are representative of all cervical cancers, our results suggest that at least 73% of cervical cancers in England are potentially preventable by type-specific protection from HPV 16/18 immunisation (i.e., are infected with HPV 16 and/or 18 only), rising to around 77% if co-infection with other HR types is never the causative HPV infection. Similarly, at least 56% and up to 65% of CIN3/CGIN are potentially preventable by HPV 16/18 immunisation. The difference between the percentage of cases with HPV 16 and/or 18 only (minimum attributable fraction), and the percentage with HPV 16 and/or 18 and another HR HPV (probable attributable fraction), indicates the potential for unmasking of disease caused by other HR HPV types if HPV 16 and 18 infections are prevented. By the same reckoning, between 32 and 49% of the moderate and 40 and 64% of the severe dyskaryotic cervical abnormalities identified by cervical screening may be preventable by HPV 16/18 immunisation.

HPV 31 and 45 were each associated (without HPV 16 and/or 18, but with or without other HR types) with an additional 3% of SCC and 2% of ADC. As there is some evidence of cross-protection from current HPV 16/18 vaccines against infection and disease due to these types and HPV 33, 52 and 58, which are closely related to HPV 16 or HPV 18 (Brown *et al*, 2009; Paavonen *et al*, 2009), the benefits of immunisation may include reduction in these cancers also. If cross-protection against HPV 31, 33, 45, 52 and 58 ranges from 25 to 29%, as suggested currently by clinical trials (Brown *et al*, 2009; Paavonen *et al*, 2009), our findings suggest that HPV 16/18 immunisation may prevent an additional 3–4% of cervical cancers in England.

Other studies of HPV type distribution in cervical cancers in the United Kingdom have reported similarly high fractions of cases probably attributable to HPV 16 and/or 18. Cuzick *et al* (2000) analysed cervical scrapes from 116 women with cervical cancer and found HPV 16 and/or 18 in 78% of SCC and 71% of ADC (including adenocarcinoma and adeno-squamous carcinoma). In Scotland, Tawfik El-Mansi *et al* (2006) found HPV 16 and/or 18 in 61% of ADC diagnosed between 1991 and 2001, and Cuschieri *et al* (2010) detected HPV 16 and/or 18 in 72% of 370 invasive cervical cancers diagnosed up to 2004. In Wales, Powell *et al* (2009) found HPV 16 and/or 18 in 80% of SCC (*N*=222) and 91% of ADC (*N*=47) diagnosed between 2000 and 2006. Three other studies have looked at fewer than 50 cases each (Crook *et al*, 1992; Arends *et al.*, 1995; Giannoudis *et al*, 1999). The differences between these United Kingdom-based studies may reflect differences in HPV typing methods, and/or chance, and do not suggest significant variations between countries in the contribution of HPV 16 and/or 18 to cervical cancer incidence that are likely to have important effect on the impact of immunisation.

Our results are consistent with previous suggestions that a higher proportion of disease in Europe will be preventable by current HPV 16/18 vaccines than some other areas of the world (Muñoz *et al*, 2004; Li *et al*, 2010), mostly because of higher HPV 16 prevalence. Furthermore, the most common non-vaccine types identified in our study (HPV 33, 45, 52 and 31) were among the most common types found in international studies, albeit not in exactly the same ranking (Clifford *et al*, 2003; Muñoz *et al*, 2004; Li *et al*, 2010).

Several studies have reported data on HPV prevalence in women attending for cervical screening in the United Kingdom (Cuschieri *et al*, 2004; Peto *et al*, 2004; Kitchener *et al*, 2006; Hibbitts *et al*, 2008). In England, HR HPV prevalence of 16% (samples collected between 2001 and 2003) (Kitchener *et al*, 2006) and 7% (samples collected between 1988 and 1993) (Peto *et al*, 2004) have previously been reported from studies conducted in Manchester. In south Wales (2008), HR HPV prevalence of 11% has been reported (Hibbitts *et al*, 2008), while in Scotland (2004), HR HPV prevalence of 16% has been reported (Cuschieri *et al*, 2004). In our study, the weighted (by age and cytology grade) prevalence of HR HPV was 16% (95% CI 11–22). These studies from across the United Kingdom also report fairly consistent results with respect to the prevalence of HPV 16 and/or 18. Differences in the age range, HPV typing methods, sample types and chance variation may account for some differences between studies. Differences may also reflect increases in prevalence over time, as the lowest HR HPV prevalence (7%) was found in the earliest study (Peto *et al*, 2004), over a period when there has been an increasing frequency of clinically apparent HPV 6 and 11 infection as cases of genital warts (Health Protection Agency, 2008).

Strengths of our study include collection of both LBC samples and biopsy samples from a number of sites geographically spread across England, and testing for the same HPV types. The stratified sampling of LBCs from each centre enabled the description of type-specific infection in more severe cytology abnormalities and from older women.

This was a cross-sectional study of a disease state that spans a long time course of progression from infection to cancer: when comparing the type distribution in cancers and in lower grade disease we cannot therefore rule out any affect from the profile of HPV types in the population changing over time. Unfortunately, data on ethnicity, deprivation and sexual behaviour were not available with our samples and therefore the association between these factors and HPV prevalence could not be analysed.

Our estimates of prevalence are based on cervical screening. As repeat screens from individuals were not excluded and may be expected to have a higher HPV prevalence, our estimates may overestimate population prevalence among screened women (as do those from other similar studies of cervical screens). Our prevalence estimates do not provide data on the 20% of the population who do not regularly attend for screening (The NHS Information Centre, 2009), and who would have more to gain from immunisation.

This is the first study to describe comprehensively type-specific HPV prevalence across England, including samples from women with normal cytology and all stages of cervical disease in order to be able to estimate the proportion of disease potentially preventable, nationally, by immunisation. These data will be used in models to assess the likely benefit from HPV immunisation (Jit *et al*, 2008; Choi *et al*, 2009), and as baseline data against which to evaluate changes in type-specific HPV prevalence and type distribution after the introduction of the HPV immunisation programme. The findings support optimism regarding high impact of the National HPV (16/18) Immunisation Programme on cervical disease in England.

## Figures and Tables

**Table 1:** Prevalence of high risk (HR) HPV types by age-band and cervical grade for the screened population (overall, by age and by disease grade) and for women with severe cervical abnormalities and cervical cancer.

		**HPV 16 and/or 18**			**HPV 16 and/or 18 without any other HR types**			**HPV 16 and/or 18 with another HR type**			**HR HPV but not HPV 16 and/or 18**			**Any HR HPV**		
	***N*** a	**%**	**(95% CI)** b	**(*n*)**	**%**	**(95% CI)** b	**(*n*)**	**%**	**(95% CI)** b	**(*n*)**	**%**	**(95% CI)** b	**(*n*)**	**%**	**(95% CI)** b	**(*n*)**
*LBC samples* c																
All screened population	4719	5.1%	(3.3–8.0)		3.2%	(2.0–5.1)		1.9%	(1.0–3.4)		10.6%	(7.2–15.3)		15.7%	(11.2–21.6)	
25–29 years	611	9.2%	(6.1–13.7)		5.2%	(3.4–7.9)		4.0%	(2.2–7.0)		19.6%	(14.4–26.1)		28.8%	(20.8–38.3)	
30–39 years	1008	6.2%	(3.2–11.8)		3.6%	(1.9–6.9)		2.6%	(1.2–5.4)		11.7%	(7.6–17.7)		17.9%	(12.1–25.8)	
40–49 years	1476	2.8%	(1.6–4.8)		2.1%	(0.8–5.3)		0.7%	(0.4–1.3)		6.8%	(5.7–8.1)		9.5%	(8.7–10.5)	
50–64 years	1463	2.7%	(1.8–3.8)		2.2%	(1.3–3.6)		0.5%	(0.2–1.3)		6.6%	(3.7–11.5)		9.3%	(6.4–13.2)	
Normal	2452	3.6%	(2.2–5.7)		2.4%	(1.4–4.1)		1.2%	(0.6–2.6)		8.6%	(5.8–12.5)		12.2%	(9.1–16.2)	
Borderline	1051	16.5%	(14.3–18.9)		9.2%	(6.8–12.4)		7.3%	(5.9–9.0)		33.2%	(27.1–40.0)		49.7%	(41.1–58.4)	
Mild dyskaryosis	697	25.6%	(21.0–30.8)		13.5%	(10.8–16.7)		12.1%	(8.6–16.8)		45.5%	(39.2–51.9)		71.1%	(61.8–78.9)	
Moderate dyskaryosis	276	49.1%	(44.1–54.1)		31.6%	(25.6–38.3)		17.4%	(12.2–24.3)		39.1%	(35.5–42.8)		88.1%	(83.9–91.4)	
Severe dyskaryosis	243	63.7%	(56.5–70.3)		39.8%	(31.1–49.2)		23.9%	(19.6–28.9)		30.3%	(22.9–38.9)		93.9%	(89.3–96.7)	
																
*Biopsies*																
CIN3	906	63.2%	(57.0–69.1)	(573)	53.9%	(48.7–59.0)	(488)	9.4%	(5.6–15.2)	(85)	30.9%	(27.4–34.6)	(280)	94.2%	(87.7–97.3)	(853)
SCC	450	76.4%	(71.0–81.1)	(344)	72.0%	(67.3–76.3)	(324)	4.4%	(2.0–9.5)	(20)	19.3%	(14.1–25.9)	(87)	95.8%	(94.4–96.8)	(431)
CGIN	54	90.7%	(85.0–94.4)	(49)	83.3%	(61.1–94.1)	(45)	7.4%	(1.2–34.5)	(4)	7.4%	(3.3–15.7)	(4)	98.1%	(86.4–99.8)	(53)
ADC	105	81.9%	(73.2–88.2)	(86)	75.2%	(64.3–83.7)	(79)	6.7%	(2.4–17.0)	(7)	13.3%	(6.1–26.7)	(14)	95.2%	(84.8–98.6)	(100)

Abbreviations: ADC=adeno and adeno-squamous carcinoma; CI=confidence interval; CIN3=cervical intraepithelial neoplasia 3; CGIN=cervical glandular intraepithelial neoplasia; HPV=human papillomavirus; HR=high-risk; LBC= liquid-based cytology; SCC=squamous cell carcinoma.

a 161 LBC samples, 133 CIN3, 2 CGIN, 5 SCC and 2 ADC from women aged <25 years.

b 95% CIs were calculated allowing for samples being clustered within laboratories.

c Weighted to allow for disproportionate LBC sample collection by age and/or cytology grade as appropriate.

**Table 2:** HR and HPV 16 and/or 18 prevalence by region (submitting laboratory) in women undergoing cervical screening

			**Prevalence**^a^ **(95% CI)**	
**Submitting laboratory**	**Sample type**	***N* (%)**	**HR HPV**	**HPV 16 and/or 18**
Birmingham (BW)	Surepath	1019 (21.6)	13.8 (11.3–16.7)	4.4 (3.1–6.2)
Gateshead (GH)	Surepath	1063 (22.5)	16.9 (14.3–19.9)	4.2 (3.0–5.9)
Gloucestershire (GL)	Thinprep	927 (19.6)	13.3 (10.4–16.9)	6.4 (4.6–6.6)
Norfolk (NN)	Thinprep	1077 (22.8)	16.2 (13.4–19.4)	6.2 (4.6–8.4)
London (RF)	Thinprep	633 (13.4)	20.1 (15.1–26.2)	6.5 (3.8–10.8)
			*P*=0.0709^b^	*P*=0.3809^b^

Abbreviations: BW=Birmingham Women’s NHS Foundation Trust; CI=confidence interval; GH=Gateshead Health NHS Foundation Trust; GL=Gloucestershire Hospitals NHS Foundation Trust; NN=Norfolk and Norwich University Hospitals NHS Foundation Trust; RF=Royal Free Hampstead NHS Trust (London).

a All prevalence estimates weighted for each laboratory to allow for differences between age and cytology distribution in study sample *vs* national population.

b Pearson's *χ*^2^.

**Table 3:** Prevalence of selected A9 and A7 types in squamous cell carcinoma (SCC) and adeno- and adeno-squamous carcinoma (ADC), with and without human papillomavirus (HPV) 16 and 18

		**SCC**					**ADC**				
**Species**	**HPV type**	**Prevalence in the absence of HPV 16 and HPV 18 (95% CI)** a		**(*n*)**	**Prevalence**	**(*n*)**	**Prevalence in the absence of HPV 16 and HPV 18 (95% CI)** a		**(*n*)**	**Prevalence**	**(*n*)**
A9	31	2.9%	(1.5–5.4)	(13)	3.1%	(14)	1.9%	(0.2–17.6)	(2)	1.9%	(2)
	33	5.3%	(3.7–7.7)	(24)	5.8%	(26)	4.8%	(1.7–12.9)	(5)	4.8%	(5)
	52	3.1%	(2.1–4.6)	(14)	3.6%	(16)	1.9%	(0.6–6.3)	(2)	3.8%	(4)
	58	0.4%	(0.1–2.2)	(2)	0.9%	(4)	0.0%	(0.0–6.6)^*^	(0)	0.0%	(0)
A7	45	3.3%	(1.6–6.6)	(15)	4.2%	(19)	1.9%	(0.6–6.3)	(2)	2.9%	(3)
	39	1.8%	(0.8–4.1)	(8)	2.2%	(10)	1.0%	(0.1–6.5)	(1)	1.9%	(2)
HPV 31, 33, 39, 45 or 52^b^		15.8%	(11.0–22.2)	(71)	18.2%	(82)	11.4%	(4.5–25.9)	(12)	15.2%	(16)
HPV 31, 33, 45, 52 or 58^c^		14.7%	(9.3–22.3)	(66)	17.1%	(77)	10.5%	(4.3–23.2)	(11)	13.3%	(14)

a 95% confidence intervals were calculated allowing for samples being clustered within laboratories except where these could not be determined because of no variation between laboratories (i.e., all 0% prevalence), when one-sided 97.5% confidence intervals have been calculated (^*^).

b Five most common types in SCC after HPV 16 and 18.

c Types against which some cross-protection efficacy results have been reported from clinical trials (Paavonen *et al*, 2009; Brown *et al*, 2009).

## APPENDIX

### Study Group Collaborators

Andrew Bailey, Henry Kitchener (Chief Investigator), Alex Sargent (Central Manchester NHS Foundation Trust); Simon Beddows, Catherine Lowndes, John Parry, Kate Soldan, (HPA); Phil Bullock (Gloucestershire Hospitals NHS Foundation Trust); Paul Cross, Martin Jones, Trudy Johnson (Gateshead Health NHS Foundation Trust); Ray Lonsdale, Richard Wood, Carol Taylor (Norfolk and Norwich University Hospitals NHS Foundation Trust); Julietta Patnick (NHSCSP); Naveena Singh (Barts and the London NHS Trust); John Smith (Sheffield Teaching Hospitals NHS Foundation Trust); Christine Waddell, Raji Ganesan, Linda Bentley, Jane Adams (Birmingham Women’s NHS Foundation Trust); Martin Young, John McGloin (Royal Free Hampstead NHS Trust).
