# Multi-site study of HPV type-specific prevalence in women with cervical cancer, intraepithelial neoplasia and normal cytology, in England

**DOI:** 10.1038/sj.bjc.6605892

**Published:** 2010-09-07

**Authors:** R Howell-Jones, A Bailey, S Beddows, A Sargent, N de Silva, G Wilson, J Anton, T Nichols, K Soldan, H Kitchener

**Correction to**: *British Journal of Cancer* (2010) **103**, 209-216; doi:10.1038/sj.bjc.6605747

Owing to an error during final correction of this paper, Figure 3 was incorrectly reproduced. The correct Figure 3 is now shown, below. The publishers apologise for this mistake.

## Figures and Tables

**Figure 3:**
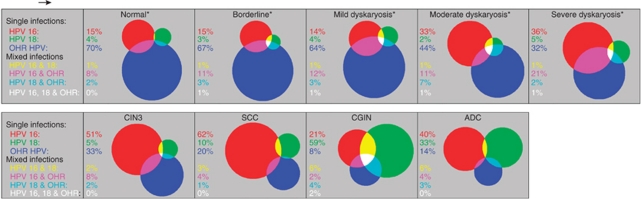
Proportional Venn diagrams showing human papillomavirus (HPV) 16, HPV 18 and high-risk (HR) types other than HPV 16 or HPV 18 (OHR) in HR HPV-positive samples, by cervical grade (Chow and Rodgers, 2005). Red: HPV 16; green: HPV 18; blue: OHR; yellow: HPV 16 and HPV 18; pink: HPV 16 and OHR; turquoise: HPV 18 and OHR; white: HPV 16, 18 and OHR. ^*^Age-weighted percentages (to allow for disproportionate liquid-based cytology sample collection by age). Abbreviations: ADC=adeno and adeno-squamous carcinoma; CIN3=cervical intraepithelial neoplasia 3; CGIN=cervical glandular intraepithelial neoplasia; SCC=squamous cell carcinoma.

