# Supplementary material for: Multi-site study of HPV type-specific prevalence in women with cervical cancer, intraepithelial neoplasia and normal cytology, in England
Source: Br J Cancer. 2010 Jul 13;103(2):209–16. doi: 10.1038/sj.bjc.6605747 (PMC2906740; doi:10.1038/sj.bjc.6605747)
Supplement: Supplementary online material [file 6605747x1.doc]

**Howell-Jones *et al*, Supplementary online material**

**Further methods – HPV genotyping**

For an assessment of assay reproducibility for HPV genotypes 6, 11, 16, 18, 26, 31, 33, 35, 39, 45, 51, 52, 53, 56, 58, 59, 66, 68, 73 and 82, we undertook a comparison of Roche LA test results derived from a single repeat testing of 198 LBC samples that had previously been included in failed test Roche LA test runs for reasons that were not thought to interfere with the analysis (for example, contamination of a negative control within a run by HPV type 62, not included within the above panel), and Roche LA test results derived from a small panel of 44 samples that were examined in three independent tests. The presence or absence of each individual HPV type listed above for each sample was scored using bands that were equal to or stronger than the β-globin low control band, or by scoring all visible bands on the genotyping strip, and a Kappa (**ĸ**) statistic was generated. For the 198 repeat samples, using only the strong type-specific band signals **ĸ** = 0.878 (IQR: 0.835 – 0.924) compared with **ĸ** = 0.800 (IQR: 0.753 – 0.895) when faint bands were also included. For the small panel of 44 samples, HPV type-specific data that were identical in all three tests (unequivocal) were compared to data which also included HPV types present in only one or two tests (unequivocal + equivocal) gave a **ĸ** = 1.000 (IQR: 0.845 – 1.000) when only strong signals were scored compared with a **ĸ** = 0.817 (IQR: 0.654 – 0.940) when faint bands were also included. For the present study, therefore, only those bands that gave a signal equal to or greater than the β-globin low control band were recorded.

Supplementary Table 1. Number of samples from each participating laboratory and HR HPV positivity.

| Submitting laboratory | Biopsies | | LBC samples | | |
| --- | --- | --- | --- | --- | --- |
|  | N | HR HPV positive | N | HR HPV positive | Sample type |
| Birmingham (BW) | 203 | 182 | 1,019 | 344 | Surepath |
| Gateshead (GH) | 324 | 313 | 1,063 | 318 | Surepath |
| Gloucestershire (GL) |  |  | 927 | 270 | Thinprep |
| London (LT) | 10 | 9 |  |  |  |
| London (RF) | 270 | 250 | 633 | 351 | Thinprep |
| Manchester (MN) | 372 | 363 |  |  |  |
| Norfolk (NN) |  |  | 1,077 | 361 | Thinprep |
| Sheffiled (SH) | 336 | 320 |  |  |  |
| Total | 1,515 | 1,437 | 4,719 | 1,644 |  |

GH: Gateshead Health NHS Foundation Trust, GL: Gloucestershire Hospitals NHS Foundation Trust, LT: Barts/The London Trust; RF: Royal Free Hampstead NHS Trust (London), MN: Central Manchester University Hospitals NHS Foundation Trust, NN: Norfolk and Norwich University Hospitals NHS Foundation Trust and SH: Royal Hallamshire Hospital Sheffield.

Supplementary Table 2. Prevalence of high-risk HPV types by age-band and cervical disease grade

| **Age-**  **band** | **Cervical disease grade^a^** | **N^b^** | **HPV 16 and/or 18** | | | **HPV 16 and/or 18 without any other HR types** | | | **HPV 16 and/or 18 with another HR type** | | | **High risk HPV but not HPV 16 and/or 18** | | | **Any high risk HPV** | | |
| --- | --- | --- | --- | --- | --- | --- | --- | --- | --- | --- | --- | --- | --- | --- | --- | --- | --- |
|  |  |  | % | (95% CI)^c^ | [n] | % | (95% CI)^c^ | [n] | % | (95% CI)^c^ | [n] | % | (95% CI)^c^ | [n] | % | (95% CI)^c^ | [n] |
| 25-  29y | Normal | 213 | 5.6% | (4.0-7.8) | [12] | 3.3% | (2.2-4.8) | [7] | 2.3% | (1.0-5.2) | [5] | 16.4% | (13.5-19.9) | [35] | 22.1% | (18.4-26.2) | [47] |
|  | Borderline | 99 | 24.2% | (16.7-33.7) | [24] | 11.1% | (6.3-18.8) | [11] | 13.1% | (6.7-24.1) | [13] | 43.4% | (36.4-50.7) | [43] | 67.7% | (57.2-76.7) | [67] |
|  | Mild dyskaryosis | 160 | 30.0% | (23.4-37.5) | [48] | 17.5% | (14.2-21.3) | [28] | 12.5% | (7.7-19.7) | [20] | 43.8% | (36.0-51.8) | [70] | 73.8% | (60.0-84.0) | [118] |
|  | Moderate dyskaryosis | 77 | 58.4% | (47.8-68.4) | [45] | 36.4% | (30.5-42.7) | [28] | 22.1% | (15.4-30.6) | [17] | 35.1% | (26.8-44.3) | [27] | 93.5% | (75.0-98.6) | [72] |
|  | Severe dyskaryosis | 62 | 62.9% | (50.7-73.7) | [39] | 37.1% | (31.7-42.9) | [23] | 25.8% | (17.0-37.2) | [16] | 33.9% | (22.5-47.5) | [21] | 96.8% | (89.1-99.1) | [60] |
|  | CIN3 | 297 | 68.4% | (58.4-76.9) | [203] | 56.9% | (47.8-65.5) | [169] | 11.4% | (7.5-17.0) | [34] | 26.3% | (19.1-35.0) | [78] | 94.6% | (87.9-97.7) | [281] |
|  | SCC | 31 | 90.3% | (69.0-97.5) | [28] | 87.1% | (63.6-96.3) | [27] | 3.2% | (0.3-24.2) | [1] | 6.5% | (0.7-39.2) | [2] | 96.8% | (75.8-99.7) | [30] |
|  | CGIN | 11 | 100% | (71.5-100)* | [11] | 90.9% | (57.7-98.7) | [10] | 9.1% | (1.3-42.3) | [1] | 0.0% | (0-28.5)* | [0] | 100% | (71.5-100)* | [11] |
|  | ADC | 6 | 83.3% | (19.4-99.0) | [5] | 50.0% | (15.6-84.4) | [3] | 33.3% | (9.6-70.2) | [2] | 0.0% | (0-45.9)* | [0] | 83.3% | (19.4-99.0) | [5] |
| 30-  39y | Normal | 449 | 4.7% | (2.4-9.0) | [21] | 2.7% | (1.3-5.2) | [12] | 2.0% | (0.9-4.4) | [9] | 9.6% | (6.3-14.2) | [43] | 14.3% | (10.0-19.9) | [64] |
|  | Borderline | 212 | 14.6% | (9.7-21.4) | [31] | 9.4% | (5.3-16.1) | [20] | 5.2% | (2.4-10.8) | [11] | 33.0% | (24.4-43.0) | [70] | 47.6% | (37.8-57.6) | [101] |
|  | Mild dyskaryosis | 187 | 23.5% | (16.7-32.1) | [44] | 12.3% | (6.7-21.4) | [23] | 11.2% | (7.4-16.8) | [21] | 47.1% | (34.5-60.0) | [88] | 70.6% | (61.7-78.1) | [132] |
|  | Moderate dyskaryosis | 78 | 41.0% | (33.3-49.2) | [32] | 29.5% | (17.1-45.9) | [23] | 11.5% | (4.6-26.0) | [9] | 47.4% | (34.2-61.0) | [37] | 88.5% | (78.0-94.3) | [69] |
|  | Severe dyskaryosis | 82 | 63.4% | (51.7-73.8) | [52] | 41.5% | (31.2-52.5) | [34] | 22.0% | (19.6-24.5) | [18] | 31.7% | (19.9-46.4) | [26] | 95.1% | (91.5-97.3) | [78] |
|  | CIN3 | 339 | 58.7% | (49.5-67.4) | [199] | 50.7% | (43.7-57.7) | [172] | 8.0% | (4.9-12.7) | [27] | 36.0% | (28.9-43.8) | [122] | 94.7% | (90.4-97.1) | [321] |
|  | SCC | 114 | 78.9% | (70.2-85.6) | [90] | 72.8% | (67.9-77.2) | [83] | 6.1% | (1.7-20.0) | [7] | 18.4% | (13.8-24.2) | [21] | 97.4% | (91.9-99.2) | [111] |
|  | CGIN | 26 | 88.5% | (84.0-91.8) | [23] | 80.8% | (54.9-93.5) | [21] | 7.7% | (0.7-49.2) | [2] | 7.7% | (2.0-25.0) | [2] | 96.2% | (75.5-99.5) | [25] |
|  | ADC | 37 | 91.9% | (81.5-96.7) | [34] | 89.2% | (69.4-96.8) | [33] | 2.7% | (0.2-27.8) | [1] | 8.1% | (3.3-18.5) | [3] | 100% | (90.5-100)* | [37] |
| 40-  49y | Normal | 842 | 1.9% | (1.0-3.7) | [16] | 1.5% | (0.6-4.0) | [13] | 0.4% | (0.2-0.7) | [3] | 5.2% | (4.4-6.2) | [44] | 7.1% | (6.1-8.3) | [60] |
|  | Borderline | 343 | 9.9% | (7.1-13.8) | [34] | 5.2% | (2.9-9.3) | [18] | 4.7% | (2.8-7.7) | [16] | 30.3% | (22.0-40.1) | [104] | 40.2% | (30.9-50.4) | [138] |
|  | Mild dyskaryosis | 156 | 21.2% | (12.3-33.9) | [33] | 11.5% | (5.2-23.7) | [18] | 9.6% | (6.3-14.4) | [15] | 47.4% | (37.7-57.4) | [74] | 68.6% | (55.8-79.0) | [107] |
|  | Moderate dyskaryosis | 79 | 41.8% | (27.9-57.0) | [33] | 38.0% | (23.6-54.8) | [30] | 3.8% | (1.6-8.7) | [3] | 40.5% | (34.1-47.2) | [32] | 82.3% | (65.9-91.8) | [65] |
|  | Severe dyskaryosis | 56 | 64.3% | (52.7-74.4) | [36] | 44.6% | (29.8-60.5) | [25] | 19.6% | (8.8-38.1) | [11] | 25.0% | (19.9-30.9) | [14] | 89.3% | (74.7-95.9) | [50] |
|  | CIN3 | 103 | 65.0% | (63.8-66.3) | [67] | 62.1% | (57.7-66.4) | [64] | 2.9% | (0.7-11.8) | [3] | 31.1% | (26.5-36.1) | [32] | 96.1% | (84.8-99.1) | [99] |
|  | SCC | 90 | 74.4% | (61.5-84.2) | [67] | 71.1% | (61.1-79.4) | [64] | 3.3% | (0.8-12.8) | [3] | 24.4% | (14.1-38.9) | [22] | 98.9% | (91.2-99.9) | [89] |
|  | CGIN | 12 | 83.3% | (72.9-90.3) | [10] | 83.3% | (72.9-90.3) | [10] | 0.0% | (0-26.5)* | [0] | 16.7% | (9.7-27.1) | [2] | 100% | (73.5-100)* | [12] |
|  | ADC | 27 | 85.2% | (50.8-97.0) | [23] | 85.2% | (50.8-97.0) | [23] | 0.0% | (0-12.8)* | [0] | 14.8% | (3.0-49.2) | [4] | 100% | (87.2-100)* | [27] |
| 50-  64y | Normal | 900 | 2.2% | (1.7-2.8) | [20] | 1.9% | (1.3-2.7) | [17] | 0.3% | (0.1-1.1) | [3] | 6.0% | (3.4-10.5) | [54] | 8.2% | (5.5-12.1) | [74] |
|  | Borderline | 351 | 10.0% | (7.7-12.8) | [35] | 6.3% | (3.7-10.5) | [22] | 3.7% | (2.0-6.6) | [13] | 18.2% | (14.8-22.3) | [64] | 28.2% | (24.4-32.3) | [99] |
|  | Mild dyskaryosis | 155 | 19.4% | (13.9-26.3) | [30] | 12.3% | (8.3-17.8) | [19] | 7.1% | (4.2-11.7) | [11] | 37.4% | (26.9-49.3) | [58] | 56.8% | (49.0-64.2) | [88] |
|  | Moderate dyskaryosis | 29 | 34.5% | (22.2-49.3) | [10] | 27.6% | (13.2-48.9) | [8] | 6.9% | (1.2-30.3) | [2] | 34.5% | (13.7-63.6) | [10] | 69.0% | (40.5-87.9) | [20] |
|  | Severe dyskaryosis | 28 | 50.0% | (31.7-68.3) | [14] | 28.6% | (14.5-48.6) | [8] | 21.4% | (10.6-38.7) | [6] | 35.7% | (23.3-50.5) | [10] | 85.7% | (65.4-95.0) | [24] |
|  | CIN3 | 32 | 28.1% | (23.1-33.7) | [9] | 21.9% | (13.4-33.6) | [7] | 6.3% | (1.1-27.8) | [2] | 56.3% | (3.5-68.3) | [18] | 84.4% | (61.2-94.9) | [27] |
|  | SCC | 121 | 67.8% | (58.9-75.5) | [82] | 65.3% | (58.2-71.8) | [79] | 2.5% | (0.6-9.4) | [3] | 25.6% | (0.1-32.0) | [31] | 93.4% | (86.1-97.0) | [113] |
|  | CGIN | 3 | 100% | (29.2-100)* | [3] | 100% | (29.2-100)* | [3] | 0.0% | (0-70.8)* | [0] | 0.0% | (0-70.8)* | [0] | 100% | (29.2-100)* | [3] |
|  | ADC | 20 | 60.0% | (37.7-78.8) | [12] | 50.0% | (41.2-58.8) | [10] | 10.0% | (1.1-52.3) | [2] | 30.0% | (10.1-62.0) | [6] | 90.0% | (65.0-97.8) | [18] |
| 65y+ | CIN3 | 2 | 50.0% | (1.3-98.7)* | [1] | 50.0% | (1.3-98.7)* | [1] | 0.0% | (0-84.2)* | [0] | 0.0% | (0-84.2)* | [0] | 50.0% | (1.3-98.7)* | [1] |
|  | SCC | 89 | 82.0% | (63.0-92.4) | [73] | 75.3% | (54.2-88.7) | [67] | 6.7% | (4.0-11.3) | [6] | 12.4% | (4.8-28.1) | [11] | 94.4% | (83.9-98.2) | [84] |
|  | ADC | 13 | 76.9% | (49.7-91.8) | [10] | 61.5% | (24.7-88.7) | [8] | 15.4% | (1.7-65.7) | [2] | 7.7% | (0.4-60.9) | [1] | 84.6% | (46.1-97.3) | [11] |

CIN3: cervical intraepithelial neoplasia 3; SCC: Squamous cell carcinoma; CGIN: cervical glandular intraepithelial neoplasia; ADC: Adeno and adeno-squamous carcinoma.

a: LBC samples classified by cytology outcome for normal, borderline and mild, moderate and severe dyskaryosis cervices. Biopsy samples classified by histology outcome for CIN3, SCC, CGIN and ADC.

b: 161 LBC samples, 133 CIN3, 2 CGIN, 5 SCC and 2 ADC from women aged <25 years.

c: 95% confidence intervals were calculated allowing for samples being clustered within laboratories except where these could not be determined due to no variation between laboratories (i.e. all 100% prevalence) or when all samples were from one laboratory (i.e. CIN3 samples from women aged 65+ years), when confidence intervals (one-sided 97.5% where appropriate) have been calculated without allowing for clustering (*).

**Supplementary** Table 3. HPV type-specific prevalence^a^ by grade of disease

| **HPV** | **Normal**  **(N=2,452)** | | **Borderline**  **(N=1,051)** | | **Mild**  **(N=697)** | | **Moderate**  **(N=276)** | | **Severe**  **(n=243)** | | **CIN3**  **(N=906)** | | **SCC**  **(N=450)** | | **CGIN**  **(N=54)** | | **ADC**  **(N=105)** | |
| --- | --- | --- | --- | --- | --- | --- | --- | --- | --- | --- | --- | --- | --- | --- | --- | --- | --- | --- |
|  | %^a^ | (95% CI)^b^ | %^a^ | (95% CI)^b^ | %^a^ | (95% CI)^b^ | %^a^ | (95% CI)^b^ | %^a^ | (95% CI)^b^ | % | (95% CI)^b^ | % | (95% CI)^b^ | % | (95% CI)^b^ | % | (95% CI)^b^ |
| 16 | 2.8 | (1.7, 4.8) | 13.4 | (11.3, 15.8) | 20.5 | (17.3, 24.2) | 41.5 | (35.2, 48.2) | 56.5 | (51.5, 61.4) | 57.3 | (49.8, 64.5) | 66.0 | (55.8, 74.9) | 29.6 | (13.7, 52.8) | 47.6 | (37.5, 58.0) |
| 18 | 0.8 | (0.4, 2.0) | 4.0 | (2.5, 6.1) | 7.2 | (4.8, 10.5) | 10.1 | (4.7, 20.2) | 9.4 | (5.2, 16.4) | 7.5 | (5.0, 11.2) | 13.1 | (8.7, 19.4) | 68.5 | (49.2, 83.0) | 40.0 | (23.1, 59.7) |
| 33 | 0.5 | (0.2, 1.1) | 3.2 | (2.5, 4.2) | 7.0 | (4.5, 10.6) | 9.7 | (5.1, 17.7) | 10.9 | (8.5, 13.9) | 10.6 | (7.0, 15.7) | 5.8 | (3.7, 8.9) | 0.0 | ( - , 6.6)* | 4.8 | (1.5, 14.3) |
| 45 | 1.1 | (0.8, 1.4) | 3.9 | (2.1, 7.2) | 5.2 | (3.1, 8.5) | 4.5 | (1.1, 17.0) | 9.4 | (6.3, 13.9) | 1.8 | (1.3, 2.4) | 4.2 | (2.3, 7.7) | 1.9 | (0.2, 18.9) | 2.9 | (1.1, 7.2) |
| 52 | 1.2 | (0.4, 3.3) | 5.3 | (4.0, 7.0) | 7.0 | (5.2, 9.5) | 9.1 | (4.0, 19.3) | 7.4 | (3.0, 14.0) | 8.6 | (6.8, 10.9) | 3.6 | (2.1, 5.9) | 1.9 | (0.2, 13.6) | 3.8 | (1.1, 11.9) |
| 31 | 1.4 | (0.8, 2.4) | 6.9 | (4.2, 11.2) | 11.7 | (8.7, 15.5) | 17.1 | (14.2, 20.4) | 14.6 | (9.5, 21.6) | 10.3 | (5.7, 17.8) | 3.1 | (1.6, 5.9) | 0.0 | ( - , 6.6)* | 1.9 | (0.1, 22.0) |
| 39 | 1.0 | (0.6, 1.9) | 6.1 | (4.0, 9.3) | 6.7 | (4.1, 10.5) | 4.1 | (3.0, 5.8) | 5.6 | (2.3, 13.2) | 1.7 | (0.7, 3.7) | 2.2 | (0.9, 5.2) | 3.7 | (0.3, 31.9) | 1.9 | (0.2, 15.2) |
| 51 | 1.3 | (0.8, 2.2) | 8.2 | (3.9, 16.2) | 11.5 | (9.3, 14.2) | 9.8 | (5.7, 16.2) | 6.4 | (3.6, 11.0) | 2.4 | (1.4, 4.1) | 1.3 | (0.7, 2.6) | 0.0 | ( - , 6.6)* | 1.9 | (0.4, 8.4) |
| 59 | 1.1 | (0.6, 2.1) | 4.1 | (3.0, 5.6) | 5.4 | (2.9, 9.8) | 4.2 | (1.5, 11.2) | 4.3 | (1.2, 14.5) | 1.5 | (1.0, 2.5) | 1.3 | (0.5, 3.5) | 5.6 | (1.1, 23.7) | 1.0 | (0.1, 8.0) |
| 35 | 0.6 | (0.2, 2.0) | 2.6 | (2.0, 3.4) | 3.2 | (2.5, 4.1) | 6.3 | (2.5, 14.9) | 3.2 | (0.6, 14.2) | 2.0 | (1.5, 2.7) | 1.1 | (0.3, 4.3) | 0.0 | ( - , 6.6)* | 1.0 | (0.0, 18.0) |
| 68 | 0.6 | (0.3, 1.2) | 2.1 | (0.9, 4.7) | 2.4 | (0.9, 6.1) | 1.3 | (0.2, 8.5) | 1.7 | (0.7, 3.7) | 0.4 | (0.1, 1.4) | 1.1 | (0.3, 3.6) | 1.9 | (0.2, 18.9) | 1.0 | (0.1, 12.0) |
| 53 | 2.2 | (1.2, 4.0) | 9.1 | (6.2, 13.2) | 13.2 | (11.6, 15.0) | 5.3 | (2, 13.3) | 4.5 | (1.9, 10.5) | 1.1 | (0.3, 3.5) | 0.9 | (0.2, 5.1) | 1.9 | (0.2, 18.0) | 1.9 | (0.2, 13.8) |
| 58 | 1.3 | (0.6, 2.9) | 4.9 | (4.0, 6.1) | 5.8 | (3.6, 9.3) | 3.9 | (1.9, 8.0) | 6.1 | (3.1, 11.5) | 4.3 | (2.6, 7.0) | 0.9 | (0.1, 5.3) | 1.9 | (0.2, 18.9) | 0.0 | ( - , 3.5)* |
| 73 | 0.8 | (0.4, 1.4) | 3.6 | (1.9, 6.9) | 4.7 | (2.6, 8.2) | 6.2 | (3.1, 12.1) | 0.8 | (0.1, 4.6) | 1.4 | (0.8, 2.6) | 0.7 | (0.1, 5.9) | 0.0 | ( - , 6.6)* | 1.0 | (0.1, 8.0) |
| 6 | 0.8 | (0.6, 1.1) | 2.1 | (1.3, 3.2) | 5.3 | (3.3, 8.4) | 3.7 | (1.1, 12.3) | 2.3 | (0.8, 6) | 0.8 | (0.5, 1.3) | 0.4 | (0.1, 1.9) | 0.0 | ( - , 6.6)* | 0.0 | ( - , 3.5)* |
| 11 | 0.2 | (0.0, 1.1) | 0.8 | (0.2, 3.1) | 0.9 | (0.2, 3.7) | 0.4 | (0.0, 7.1) | 0.0 | - | 0.3 | (0.1, 0.9) | 0.4 | (0.1, 1.5) | 0.0 | ( - , 6.6)* | 0.0 | ( - , 3.5)* |
| 56 | 0.8 | (0.5, 1.6) | 5.1 | (3.8, 6.8) | 10.5 | (7.8, 14.1) | 3.9 | (1.6, 8.8) | 3.0 | (1.9, 4.7) | 0.4 | (0.1, 1.5) | 0.4 | (0.1, 1.9) | 0.0 | ( - , 6.6)* | 0.0 | ( - , 3.5)* |
| 66 | 1.3 | (0.5, 3.2) | 6.2 | (3.9, 9.7) | 10.7 | (9.0, 12.8) | 4.1 | (2.2, 7.5) | 2.7 | (0.6, 11.5) | 1.0 | (0.4, 2.4) | 0.4 | (0.0, 4.0) | 0.0 | ( - , 6.6)* | 0.0 | ( - , 3.5)* |
| 70 | 1.4 | (0.7, 2.8) | 4.5 | (2.4, 8.4) | 4.9 | (3.8, 6.2) | 4.1 | (0.9, 16.7) | 1.8 | (0.8, 3.7) | 0.3 | (0.1, 1.0) | 0.4 | (0.1, 2.2) | 0.0 | ( - , 6.6)* | 0.0 | ( - , 3.5)* |
| 42 | 1.4 | (0.9, 2.2) | 11.0 | (8.2, 14.5) | 13.2 | (9, 19.0) | 4.5 | (1.3, 14.0) | 3.1 | (1.0, 9.5) | 0.0 | ( - , 0.4)* | 0.2 | (0.0, 2.6) | 0.0 | ( - , 6.6)* | 0.0 | ( - , 3.5)* |
| 62 | 2.3 | (1.2, 4.5) | 7.8 | (4.3, 13.7) | 8.2 | (5.5, 12.0) | 7.8 | (4.3, 13.6) | 4.5 | (2.2, 8.9) | 0.3 | (0.1, 0.9) | 0.2 | (0.0, 2.0) | 1.9 | (0.2, 18.0) | 1.0 | (0.1, 9.6) |
| CP6108 | 2.0 | (0.8, 5.1) | 5.1 | (2.8, 9.1) | 8.4 | (4.1, 16.6) | 6.7 | (3.6, 11.9) | 5.1 | (1.6, 15.5) | 1.0 | (0.3, 3.3) | 0.2 | (0.0, 2.0) | 0.0 | ( - , 6.6)* | 0.0 | ( - , 3.5)* |
| IS39 | 0.0 | - | 1.1 | (0.2, 5) | 0.5 | (0.2, 1.4) | 0.0 | - | 0.0 | - | 0.8 | (0.3, 2.0) | 0.2 | (0.0, 2.6) | 0.0 | ( - , 6.6)* | 0.0 | ( - , 3.5)* |
| 26 | 0.1 | (0.0, 0.5) | 0.5 | (0.2, 1.8) | 0.2 | (0.0, 4.9) | 0.4 | (0.0, 6.3) | 0.9 | (0.2, 4.9) | 0.1 | (0.0, 1.3) | 0.0 | ( - , 0.8)* | 0.0 | ( - , 6.6)* | 0.0 | ( - , 3.5)* |
| 54 | 2.0 | (1.6, 2.5) | 3.2 | (1.8, 5.7) | 6.8 | (4.8, 9.6) | 6.5 | (2.6, 15.1) | 7.1 | (2.6, 18.1) | 0.4 | (0.1, 1.4) | 0.0 | ( - , 0.8)* | 0.0 | ( - , 6.6)* | 0.0 | ( - , 3.5)* |
| 61 | 2.5 | (1.6, 3.8) | 8.2 | (6.0, 11.0) | 7.6 | (6.1, 9.4) | 6.9 | (3.8, 12.2) | 4.8 | (1.2, 17.4) | 1.0 | (0.4, 2.3) | 0.0 | ( - , 0.8)* | 0.0 | ( - , 6.6)* | 0.0 | ( - , 3.5)* |
| 67 | 0.3 | (0.1, 0.7) | 1.5 | (0.6, 4.2) | 3 | (2.1, 4.2) | 1.3 | (0.4, 4.3) | 0.7 | (0.1, 4.7) | 0.2 | (0.0, 2.7) | 0.0 | ( - , 0.8)* | 0.0 | ( - , 6.6)* | 0.0 | ( - , 3.5)* |
| 69 | 0.1 | (0.0, 2.0) | 0.0 | - | 0.4 | (0.0, 4.9) | 0.5 | (0.1, 2.5) | 0.0 | - | 0.3 | (0.1, 1.9) | 0.0 | ( - , 0.8)* | 0.0 | ( - , 6.6)* | 0.0 | ( - , 3.5)* |
| 71 | 0.1 | (0.0, 0.9) | 0.5 | (0.1, 1.6) | 0.3 | (0.1, 1.4) | 0.4 | (0.0, 6.7) | 0.5 | (0.0, 7.2) | 0.1 | (0.0, 1.4) | 0.0 | ( - , 0.8)* | 0.0 | ( - , 6.6)* | 0.0 | ( - , 3.5)* |
| 72 | 0.3 | (0.1, 1.0) | 0.4 | (0.1, 2.0) | 1.8 | (0.8, 4.1) | 0.7 | (0.0, 11.3) | 0.5 | (0.0, 7.2) | 0.1 | (0.0, 1.4) | 0.0 | ( - , 0.8)* | 0.0 | ( - , 6.6)* | 0.0 | ( - , 3.5)* |
| 81 | 0.5 | (0.2, 1.2) | 3.7 | (2.8, 4.7) | 3.7 | (2.3, 6.0) | 1.9 | (0.7, 5.5) | 0.7 | (0.0, 10.3) | 0.2 | (0.0, 1.1) | 0.0 | ( - , 0.8)* | 0.0 | ( - , 6.6)* | 0.0 | ( - , 3.5)* |
| 82 | 0.3 | (0.1, 1.3) | 1.4 | (0.5, 3.7) | 1.8 | (0.8, 4.0) | 3.1 | (1.8, 5.0) | 2.6 | (1.1, 5.7) | 0.7 | (0.3, 1.5) | 0.0 | ( - , 0.8)* | 0.0 | ( - , 6.6)* | 0.0 | ( - , 3.5)* |
| 83 | 1.0 | (0.5, 1.9) | 2.6 | (1.4, 5.0) | 3.1 | (1.8, 5.2) | 3.0 | (1.3, 6.6) | 2.6 | (0.3, 16.9) | 0.1 | (0.0, 1.4) | 0.0 | ( - , 0.8)* | 0.0 | ( - , 6.6)* | 0.0 | ( - , 3.5)* |
| 84 | 1.3 | (0.9, 1.9) | 5.6 | (4.2, 7.5) | 6.3 | (4.8, 8.3) | 4.6 | (1.6, 12.2) | 1.3 | (0.4, 4.3) | 0.2 | (0.0, 1.1) | 0.0 | ( - , 0.8)* | 0.0 | ( - , 6.6)* | 0.0 | ( - , 3.5)* |
| 55 | 0.7 | (0.3, 1.8) | 1.7 | (0.8, 3.6) | 3.7 | (1.6, 8.5) | 2.6 | (1.1, 5.9) | 1.5 | (0.1, 20.0) | 0.0 | ( - ,0.4)* | 0.0 | ( - , 0.8)* | 0.0 | ( - , 6.6)* | 0.0 | ( - , 3.5)* |
| 40 | 0.3 | (0.1, 0.6) | 1.1 | (0.4, 2.7) | 1.4 | (0.6, 3.3) | 0.4 | (0.0, 6.7) | 0.5 | (0.0, 7.2) | 0.0 | ( - ,0.4) | 0.0 | ( - , 0.8)* | 0.0 | ( - , 6.6)* | 0.0 | ( - , 3.5)* |
| 64 | 0.0 | - | 0.1 | (0.0, 1.9) | 0.2 | (0.0, 4.4) | 0.2 | (0.0, 3.4) | 0.0 | - | 0.0 | ( - ,0.4) | 0.0 | ( - , 0.8)* | 0.0 | ( - , 6.6)* | 0.0 | ( - , 3.5)* |

Note: Types that were not detected in any samples: HPV 32, 43, 44, 57 and 74.

^a^ Weighted to allow for disproportionate LBC sample collection by age. **^b^** 95% confidence intervals (CI) were calculated allowing for samples being clustered within laboratories except where these could not be determined due to no variation between laboratories (i.e. all 100% prevalence) or when all samples were from one laboratory (i.e. CIN3 samples from women aged 65+ years). Where CIs allowing for clustering could not be determined for CIN3, SCC, CGIN and ADC samples confidence intervals (one-sided 97.5% where appropriate) have been calculated without allowing for clustering (*).
